# Role of autophagy on cancer immune escape

**DOI:** 10.1186/s12964-021-00769-0

**Published:** 2021-09-07

**Authors:** Yalan Duan, Xiaoqing Tian, Qian Liu, Jianhua Jin, Juanjuan Shi, Yongzhong Hou

**Affiliations:** 1 Department of Oncology, The Affiliated Wujin Hospital, Jiangsu University, Changzhou, 213017 Jiangsu Province China; 2 School of Life Sciences, Jiangsu University, Zhenjiang, 213017 Jiangsu Province China; 3 Department of Oncology, The Wujin Clinical College of Xuzhou Medical University, Changzhou, 213017 Jiangsu Province China

**Keywords:** Autophagy, Immune cells, Immune escape, Antigen presentation, Cancer therapy

## Abstract

Autophagy is catabolic process by degradation of intracellular components in lysosome including proteins, lipids, and mitochondria in response to nutrient deficiency or stress such as hypoxia or chemotherapy. Increasing evidence suggests that autophagy could induce immune checkpoint proteins (PD-L1, MHC-I/II) degradation of cancer cells, which play an important role in regulating cancer cell immune escape. In addition to autophagic degradation of immune checkpoint proteins, autophagy induction in immune cells (macrophages, dendritic cells) manipulates antigen presentation and T cell activity. These reports suggest that autophagy could negatively or positively regulate cancer cell immune escape by immune checkpoint protein and antigens degradation, cytokines release, antigens generation. These controversial phenomenon of autophagy on cancer cell immune evasion may be derived from different experimental context or models. In addition, autophagy maybe exhibit a role in regulating host excessive immune response. So rational combination with autophagy could enhance the efficacy of cancer immunotherapy. In this review, the current progress of autophagy on cancer immune escape is discussed.

**Video Abstract**

**Supplementary Information:**

The online version contains supplementary material available at 10.1186/s12964-021-00769-0.

## Background

Autophagy is the intracellular components (proteins, lipids, mitochondria, nucleus etc.) degrading process in lysosome in response to stressful conditions such as nutrition deficiency, hypoxia, and chemotherapy, which is also nutrients recycling [1]. Autophagy contains three types including macroautophagy, microautophagy, and chaperone-mediated autophagy (CMA). Macroautophagy process undergoes initiation, nucleation, vesicle expansion, maturation of autophagosome, fusion of autophagosome-lysosome and finally degradation of components in lysosome [2–4]. In microautophagy process, proteins and organelles are degraded by direct engulfment of lysosomes [4]. In CMA process, proteins with KFERQ motif are recognized by HSC70 (heat shock cognate 70 kDa protein) resulting in targeted proteins degradation in lysosome by LAMP2A ( lysosomal-associated membrane protein 2A) [5]. Autophagy (hereafter referred to as macroautophagy) degrades misfolded proteins or dysfunctional organelles to maintain cellular homeostasis [1]. The autophagy receptors such as p62 (SQSTM1) and NBR1 (next to *BRCA1* gene 1 protein) mediate ubiquitinated proteins for autophagic degradation leading to clearance of misfolded proteins [6, 7]. In addition, PINK1 induces ubiquitin phosphorylation leading to activation of PARKIN ubiquitin ligase, consequently facilitates ubiquitination of mitochondrial outer membrane proteins resulting in autophagy receptors (NDP52 and optineurin)-mediated damaged mitochondrial degradation [8]. Dysregulation of autophagy has been implicated in cance [9–12]. Although autophagy promotes cancer cell survival under nutrient and oxygen deprivation by degrading a bulk of organelles, proteins, and lipids for nutrients recycling [10, 11, 13–16], the role of autophagy in cancer progression is dependent on the conditions such as the tumor types and tumor models used [9–11]. Furthermore, autophagy plays an important role in regulating cancer immunotherapy by degrading immune checkpoint proteins [17, 18]. Increasing clinical evidence shows that immunotherapy is an exciting benefit for variety of tumors, while it exhibits low response rates for patients, suggesting that cancer immunotherapy is so complicated and the mechanisms could be associated with cancer types and individual difference [19]. Similarly, autophagy could negatively or positively regulate cancer immunotherapy by degradation of immune checkpoint protein, pro-inflammatory cytokines release, and antigens generation or degradation [17, 18, 20–26]. For example, in cancer cells, induction of PD-L1 autophagic degradation promotes T cell killing of cancer cells and enhances the efficacy of cancer immunotherapy [17], whereas MHC-I undergoes autophagic degradation in pancreatic ductal adenocarcinoma (PDAC) leading to loss of antigen presentation to T cells, consequently inhibits cancer immunotherapy [18]. However, the contradictory role of autophagy on cancer cell immune evasion may be derived from different experimental context or models. Autophagy maybe exhibit a role in regulating host excessive immune response. In this review, we discussed the current progress of autophagy on cancer immune escape.

### Autophagy regulates PD-1/PD-L1 immune checkpoint pathway

**Fig. 1:**
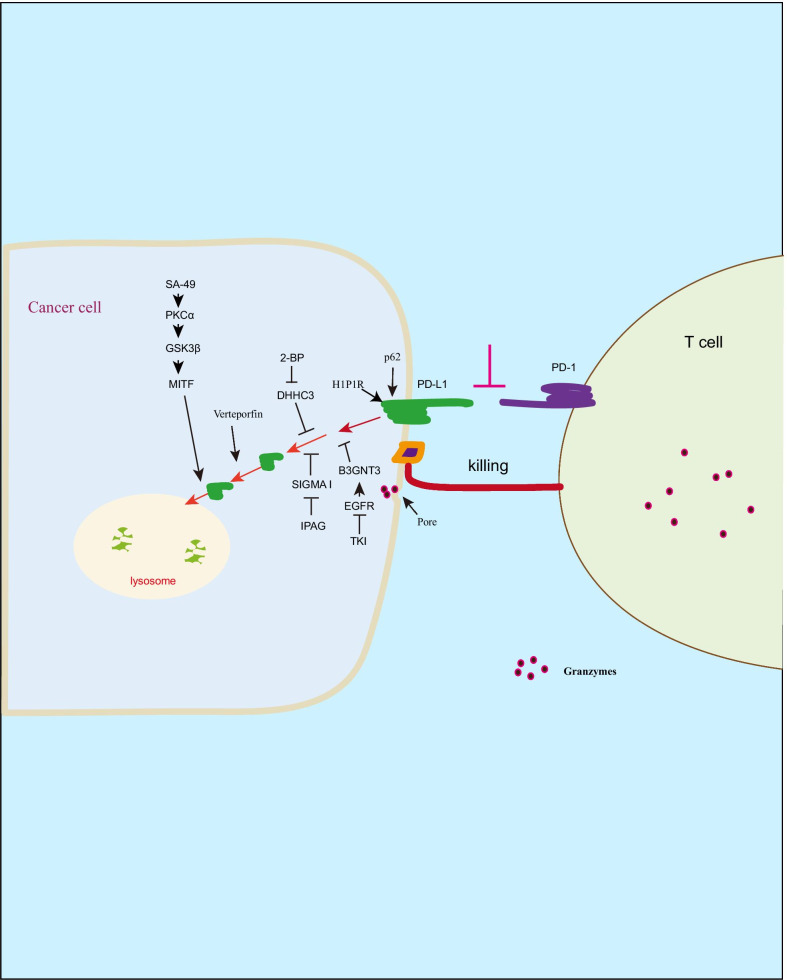
Autophagy regulates PD-1/PD-L1 immune checkpoint pathway. The binding of PD-L1 to PD-1 suppresses T cell killing to cancer cells, while PD-L1 undergoes selectively autophagic degradation by H1P1R or p62. In addition, autophagy could be activated by extracellular stimuli such as SA-49, 2-BP, veteporfin resulting in PD-L1 autophagic degradation in cancer cells, consequently, enhances T cell activity and inhibits tumor growth

Expression of PD-L1 on cancer cells binds to PD-1 on T cells leading to inhibition of T cell activation and proliferation, consequently promotes cancer immune escape [27, 28]. Therefore, PD-1/PD-L1 immune checkpoint blockade can enhance the efficacy of cancer immunotherapy 29, 30. Although cancer cell exhibits immune evasion by abnormal expression of PD-L1 [30, 31], deficiency of HIP1R in cancer cells increases PD-L1 protein levels [32]. In this study, it shows that HIP1R acts as a autophagy receptor for PD-L1 binding and induces PD-L1 selective autophagic degradation in lysosome, subsequently, inhibits tumor growth by increasing T cell cytotoxicity, suggesting that autophagic degradation of PD-L1 suppresses cancer immune escape. However, cancer cell exhibits ability to inhibit PD-L1 autophagic degradation by transcriptional modification [33, 34]. EGFR/B3GNT3 pathway-induced PD-L1 glycosylation leads to inhibition of PD-L1 autophagic degradation, subsequently, facilitates tumor immune escape in a breast xenograft tumor model [33]. PD-L1 palmitoylation by DHHC3 reduces PD-L1 endosomal sorting-mediated autophagic degradation consequent immune suppression and tumor growth in a colon tumor model [34]. In addition to PD-L1 protein modification, the cell membrane CMTM6 binds to PD-L1 consequent inhibition of endocytosed PD-L1 degradation and tumor immune evasion [35]. Therefore, in response to extracellular stimuli, activation of autophagy induces PD-L1 degradation in lysosome, subsequently, increases the efficacy of cancer immunotherapy [34, 36, 37]. SA-49 treatment facilitates PKCα/GSK3β/MITF-mediated PD-L1 autophagic degradation [36], and DHHC3 inhibitor 2-bromopalmitate (2-BP) abolishes PD-L1 palmitoylation resulting in PD-L1 autophagic degradation [34], subsequently, enhances the efficacy of cancer immunotherapy in a colon tumor model. Moreover, verteporfin induces PD-L1 degradation in Golgi-related autophagy pathway consequent T cell activation [38]. As a phase III trial drug, sunitinib promotes p62-mediated PD-L1 autophagic degradation resulting in increased anti-tumor immune response [39]. Since the interaction of SIGMA I with glycosylated PD-L1 leads to inhibition of PD-L1 autophagic degradation in breast and prostate cancer cells, SIGMA I inhibitor IPAG reverses this event leading to increased T cell activity [37]. In addition to autophagy induction by extracellular stimuli, PD-L1 blockade by PD-L1 antibody H1A inhibits the interaction of PD-L1 with CMTM6 leading to PD-L1 autophagic degradation [40]. These findings suggest that autophagy induction causes selective PD-L1 autophagic degradation, subsequently, increases T cell activity (Fig. 1). However, another study suggests that autophagy activation increases PD-L1 expression by 5-HT1aR/autophagy/p-STAT3 pathway in lung adenocarcinoma patients with depression leading to immune escape [41]. Similarly, pharmacological inhibition of PIK3C3/VPS34-mediated autophagy increases the efficacy of immunotherapy by PD-1/PD-L1 immune checkpoint blockade [42]. These findings suggest that rational PD-L1 levels could improve PD-1/PD-L1 blockade therapy.

### Autophagy and MHC-I/MHC-II

**Fig. 2:**
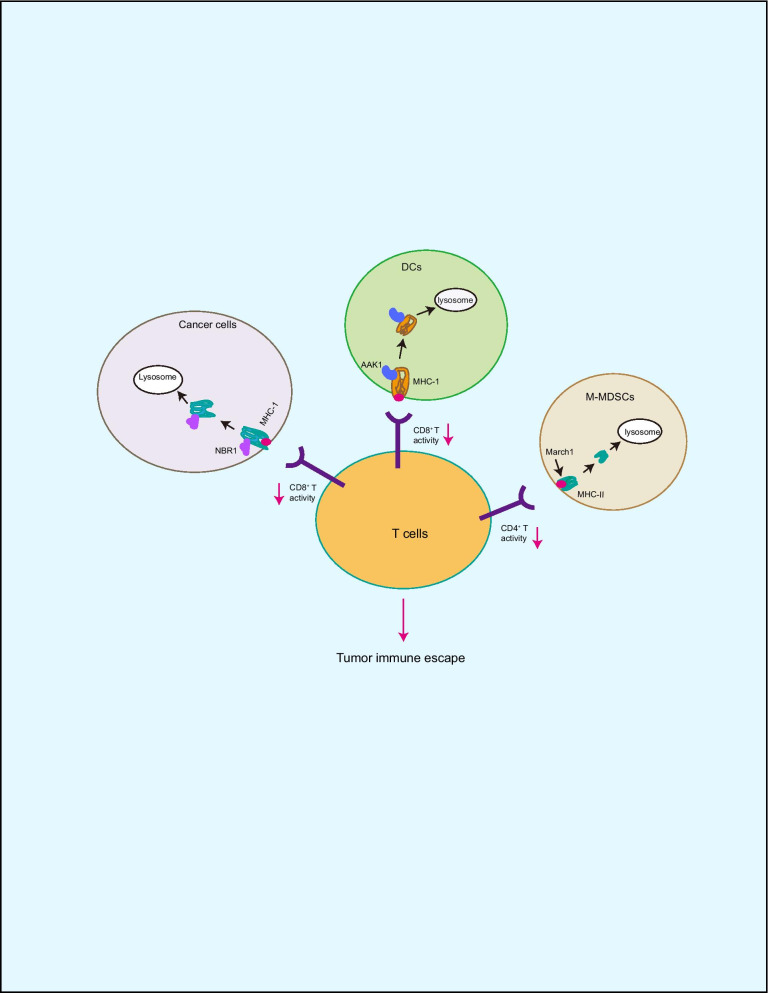
Autophagy regulates MHC-I/II stability. MHC-1/II plays a critical role in antigen presentation for T cell activation and killing, while MHC-I/II undergoes autophagic degradation in cancer cells by NBR1 or March1 leading to tumor immune escape. In addition, AAK1 induces MHC-1 autophagic degradation in DCs resulting in inhibition of antigen presentation and T cell activation

Activation of innate and adaptive immune response is critical for killing to cancer cells in host immune system [43, 44]. In this process, MHC-I/II plays an important role on antigen presenting cells (APCs) by presenting antigens to T cells consequent T cell activation [43, 44]. However, cancer cell can escape immune surveillance by degrading MHC-1 [18]. In pancreatic ductal adenocarcinoma (PDAC), MHC-I proteins are selective degradation by NBR1, subsequently, reduces antigen presentation and T cell killing to cancer cells. In contrast, autophagy inhibitors treatment enhances the efficacy of anti-tumor therapy [18]. Similarly, March1 E3 ubiquitin ligase induces MHC-II autophagic degradation in M-MDSCs (myeloid-derived suppressor cells) leading to cancer immune evasion. Conversely, autophagy inhibition by ATG5 deficiency elevates cell surface MHC-II protein levels leading to increased CD4^+^ T cell activation [45]. Although one study shows that autophagy activation in response to irradiation increases MHC-I expression and CD8^+^ T cell infiltration in NSCLC cells, the direct relationship of MCH-1 expression with autophagy is unclear [46]. In addition to degradation of MHC-1 in cancer cells, AAK1 mediates MHC-1 endocytosis and autophagic degradation in DCs resulting in inhibition of antigen presentation and CD8^+^ T cell priming, which is reversed in DCs by deficiency of autophagy [47]. These findings suggest that autophagic degradation of MHC-I/II in cancer cells facilitates tumor immune evasion (Fig. 2). However, in the tumor microenvironment, could cancer cell regulate MHC-1 autophagic degradation in DCs?

### Mitophagy and tumor immune escape

**Fig. 3:**
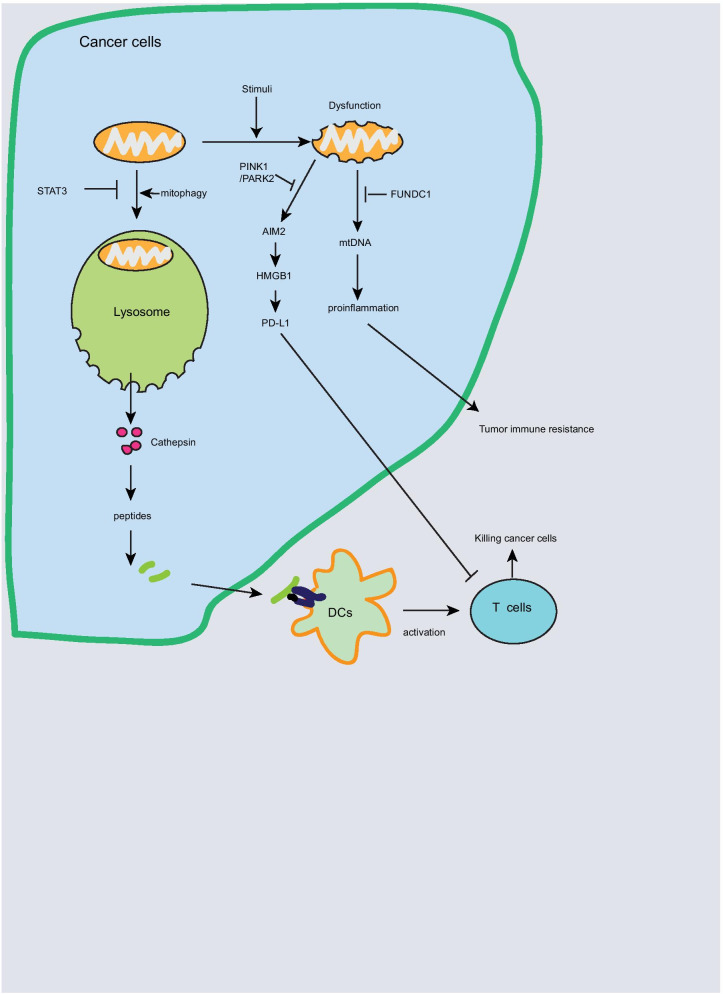
Mitophagy and tumor immune escape. Mitophagy plays an important role in regulating immune response against cancer. Mitophagy induction in STAT3 deleted cancer cells increases antigen presentation for DCs and T cell activation. In addition, PINK1/PARK2 or FUNDC1-mediated mitophagy promotes clearance of damaged mitochondria leading to increased antitumor immune response

Mitophagy is a selective autophagy process by clearance of damaged or dysfunctional mitochondria, which is triggered in response to stimuli such as hypoxia, DNA damage, and nutrient starvation [48]. Mitophagy plays an important role in regulating immune response against cancer [49–51]. Increased mitophagy in STAT3 deleted intestinal epithelial cells facilitates lysosomal membrane permeabilization by increasing Fe^2+^ accumulation, which in turn releases cathepsin into cytosol and generates peptides for antigen presentation, leading to T cell activation and anti-tumor immunity [49]. PINK1/PARK2 pathway-mediated mitophagy is essential for clearance of damaged mitochondria and inhibits tumor development [50]. Deficiency of *Pink1* and *Park2* promotes pancreatic tumorigenesis in Kras-driven tumor model by increasing mitochondrial iron accumulation and AIM2/HMGB1 pathway-mediated PD-L1 expression [51]. As a mitophagy receptor, FUN14 domain-containing 1 (FUNDC1)-mediated mitophagy inhibits hepatocellular carcinoma (HCC) initiation and progression in response to diethylnitrosamine, whereas hepatocyte-specific FUNDC1 deficiency increases dysfunctional mitochondria accumulation and cytosolic mitochondrial DNA (mtDNA) release, which in turn promotes proliferation of hepatocytes by pro-inflammatory response [52]. Cytosolic mitochondrial DNA stress activates TLR9/NFκB/CCL2 pathway, and then increases TAM (tumor-associated macrophage)-induced HCC [53]. These findings suggest that damaged mitochondrial clearance or lysosomal membrane permeabilization-mediated antigen presentation could enhance anti-tumor immune response (Fig. 3), which contributes to cancer immunotherapy. However, mitophagy inhibition in NLRX1 deficiency inhibits turnover of damaged mitochondria in response to TNF-α, leading to inhibition of oxidative phosphorylation (OxPhos)-dependent triple-negative breast cancer cell proliferation and migration [54]. In contrast to mitophagy promotes antigen presentation in colorectal cancer (CRC) [49], in response to LPS or heat stress, PINK1 and PARK2 inhibits mitochondrial antigen presentation [55]. These discrepancy phenomenon may be derived from different model and conditions.

### Autophagy, exosome and immune escape

**Fig. 4:**
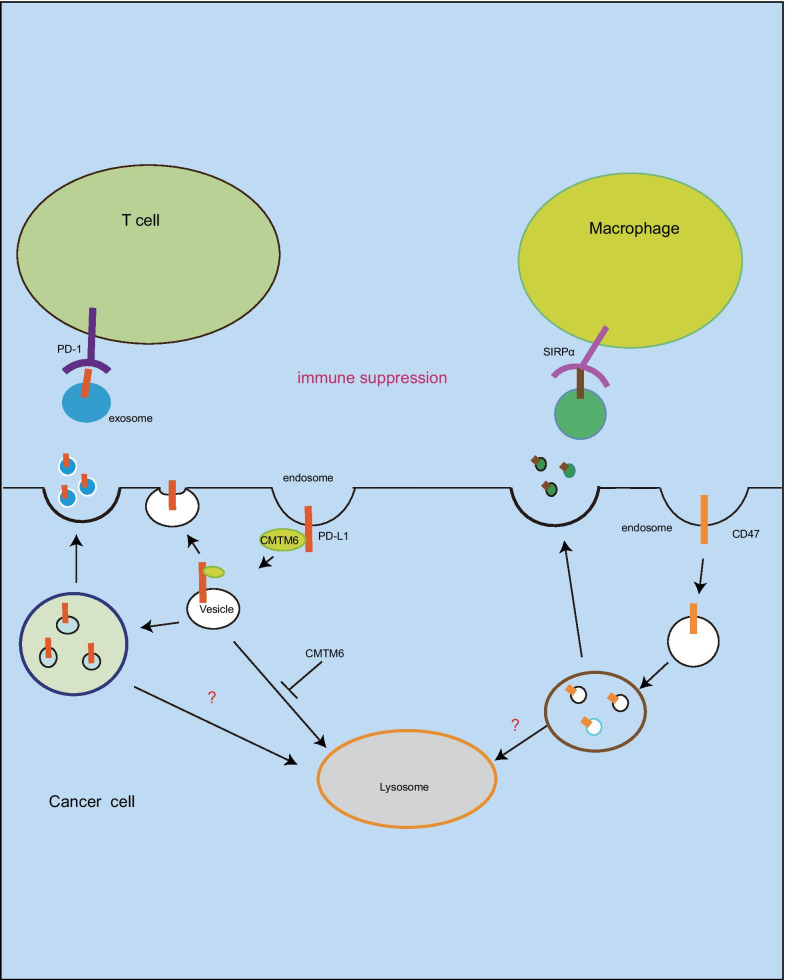
Autophagy, exosome and immune escape. PD-L1 undergoes endosome and degradation in lysosome, but CMTM6 facilitates endocytosed PD-L1 recycling to membrane. The immune checkpoint protein PD-L1 and CD47 are presented on exosomes, which maybe escape autophagic degradation in lysosome

Exosomes are cellular secreted vesicles (30–150 nm) with double-layer membrane, which play an important role in regulating crosstalk between cells [56]. Exosomes are generated from endosome-derived multivesicular bodies (MVBs) without degradation by lysosomes [57]. The membrane PD-L1 protein undergoes endosome cycling, and exosomal PD-L1 is observed in multiple types of cancer cells [58]. Tu et al. have described that the binding of membrane CMTM6 to PD-L1 is required for PD-L1 trafficking to cell surface rather than autophagic degradation in lysosome [40]. In addition to PD-L1 releases extracellular by exosome pathway, CD47 is present on exosome [59–61]. SIRPα (signal-regulatory protein α)/CD47 immune checkpoint is “don’t eat me” signal [62, 63], which inhibits phagocytosis of cancer cells by macrophage [62–64]. CD47 is highly expressed on cancer cells [65, 66]. The binding of CD47 to the surface SIRPα on macrophage resulting in inhibition of phagocytosis [62, 65]. Exosomal CD47 decreases pancreatic cancer cell clearance by phagocytes [61], while the relationship of exosomal CD47 with autophagy is still unclear. These findings suggest that autophagy induction could contribute to cancer immunotherapy by PD-L1 or CD47 degradation in lysosome (Fig. 4).

### Autophagy negatively or positively regulates immune response against cancer cells

**Fig. 5:**
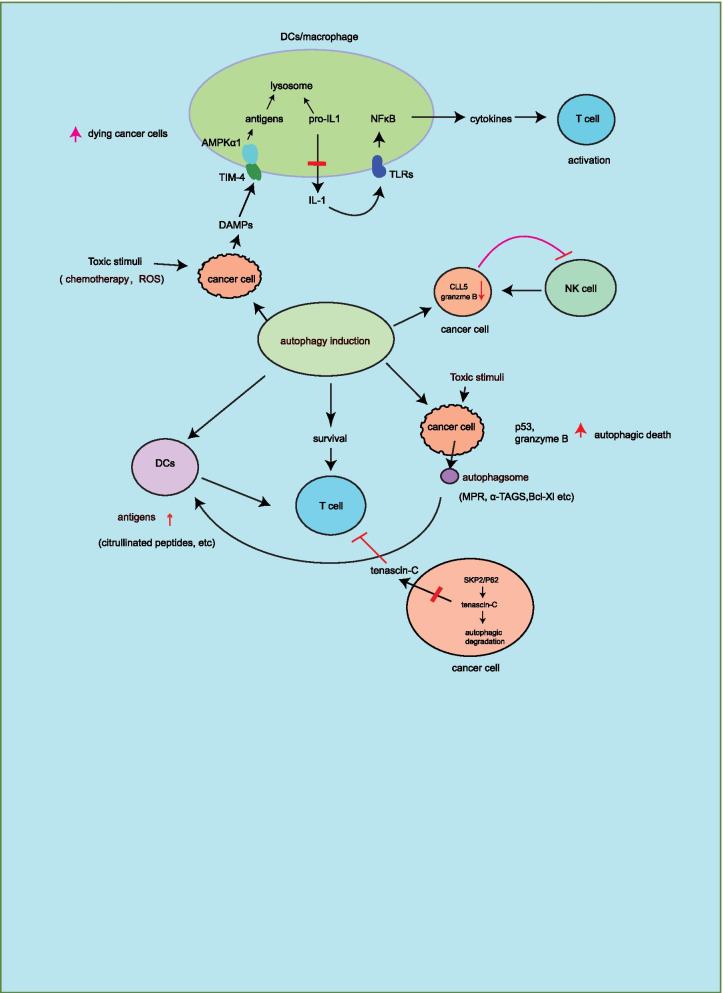
Autophagy positively or negatively regulates immune response against cancer. Autophagy induction acts as inhibition or promotion of cancer immune escape. This controversial phenomenon may be derived from the experimental context. Some experimental models are in vitro analysis, whereas the regulatory role of autophagy on cancer immune response is complicate in the tumor microenvironment in response to hypoxia, cytokines, or chemotherapy. Autophagy maybe exhibit synergistic effect with immune cells on regulation of cancer immune surveillance

Autophagy promotes T cell proliferation and survival, which exhibits ability to maintain ER homeostasis by regulating intracellular calcium stores in T cells, in contrast, autophagy inhibition by deleted ATG5 results in T cell death [67]. Increasing evidence suggests that autophagy induction enhances antigen generation and presentation [20–23]. Activation of autophagy increases antigen presentation of DCs and T cell priming leading to inhibition of tumor growth [20]. In the antigen presenting cells (APCs), autophagy induction generates citrullinated peptides that are antigen presentation of MHC-II on CD4^+^ T cells [21]. In response to radiation therapy, autophagy causes loss of its natural ligands of MPR (mannose-6-phosphate receptor), which is transferred to cell surface leading to increased T cell killing and CTLA4 antibody immunotherapy in B16F10-bearing tumor model [22]. α-TEA (α-tocopheryloxyacetic acid) induces autophagic death in lung cancer cells, and then the release of autophagosome with α-TAGS acts as an effective antigen presentation on DCs, subsequently, increases CD8^+^ T cell killing to cancer cells [23]. Autophagy activation in response to temozolomide and VPA in GL261 glioma cells increases T cell activity [68]. As a secreted cellular matrix protein, tenascin-C inhibits T cell activation, but SKP2 induces tenascin-C ubiquitination and p62-mediated autophagic degradation, in contrast, autophagy deficiency reverses this event resulting in (TNBC) triple-negative breast cancer resistance to T cell [69]. In addition to T cell activation by autophagy, autophagy induction also increases NK cell killing by HMBOX1 (homeobox containing 1) [70] in HepG2 cells or activation of p53 in breast cancer cells [71]. Chollat-Namy et al. [71] have described that pharmacological reactivation of mutant p53 by CP31398 results in autophagy induction in breast cancer cells. Mechanistically, CP31398 blocks the infusion of lysosome and autophagosome that contains anti-apoptotic Bcl-Xl, XIAP proteins. In addition, this drug also inhibits granzyme B degradation that is important for NK cell killing. However, activation of mutant p53 by CP31398 is involved in multiple pathways, which maybe have alternative mechanism that is independent of p53 function for NK activation. These findings suggest that autophagy induction positively regulates immune response against cancer cells (Fig. 5).

In contrast to activation of immune response by autophagy induction, some reports suggest that autophagy induction inhibits T cell activation in response to EMT (epithelial-to-mesenchymal transition) [72], ROS (reactive oxygen species) [73], and chemotherapy [24, 25] leading to impaired T cell killing and promotion of tumor growth, which is associated with autophagy-mediated antigen degradation [24, 25] and inhibition of DCs activity [73] (Fig. 5). Loss of antigen presentation by APCs impairs T cells priming [74], thus activation of autophagy in macrophages or DCs promotes antigen degradation in lysosome, consequently impairs T cell killing and promotes MC38 colon cancer growth [24]. Mechanistically, the release of DAMPs (danger-associated molecular patterns) from chemotherapy-induced dying cancer cells increases TIM-4 expression and surface distribution on macrophages and DCs, and then TIM-4 binds to AMPKα1 consequent autophagy induction and antigen degradation in lysosome. Consistently, in response to chemotherapy, treatment with autophagy inhibitor (chloroquine) effectively increases CD8^+^ T cell killing to colon cancer cells [25] and CD4^+^ T cell killing to lung cancer cells [75]. Activation of STING-mediated pro-inflammatory cytokine release could facilitate T cell priming [74]. Therefore, another report shows that SKIL/TAZ-induced autophagy inhibits STING pathway-mediated antitumor immune response [76]. In this study, it shows that SKIL increases TAZ protein stability by inhibition of LATS2 activity, which in turn promotes autophagy and tumorigenesis of lung cancer. In addition, SKIL/TAZ/autophagy pathway reduces pro-inflammatory cytokine release including CXCL10, CCL5, and IFN-β, which could be activation of STING pathway-mediated antitumor immune response, but the direct relationship of autophagy in NSCLCs with STING pathway is unclear. Autophagy induction reduces IL-1β release [77], which in turn inhibits IL-1/TLR/NFκB-mediated pro-inflammatory cytokine release in macrophages and DCs resulting in impaired γδ T cell activation [26]. In contrast, combined chloroquine (autophagy inhibitor) with IL-2 increases IL-1 immunotherapy in metastatic liver tumor model [78]. These findings suggest that autophagy inhibits pro-inflammatory response-mediated antitumor immune therapy. In addition to inhibition of T cell activity by autophagy, deficiency of autophagy promotes CLL5 expression resulting in NK cell infiltration and tumor growth inhibition of melanomas [79]. In response to hypoxia, autophagy induction causes resistant to NK cell killing by granzyme B autophagic degradation in breast cancer cells [80]. These reports suggest that autophagy induction in APCs or cancer cells impairs immune cell activity such as DCs, T cells and NK cells, which are associated with antigen presentation or granzyme B degradation. These findings suggest autophagy induction negatively regulates immune response against cancer cells (Fig. 5).

## Conclusion

Autophagy induction could enhance effectivity of cancer immune therapy by PD-L1 autophagic degradation in multiple types of cancer cells [17, 34, 36, 37, 39]. Conversely, NBR1 induces MHC-I selective autophagic degradation in PDAC consequent tumor immune escape, while autophagy inhibitor treatment increases the efficiency of anti-tumor immune therapy [18]. Since PD-L1 undergoes endosome trafficking and autophagic degradation in lysosome [35], does autophagy inhibition in PDAC could elevate PD-L1 protein levels? Moreover, internalized CTLA-4 (cytotoxic T-lymphocyte antige 4) immune checkpoint on T cells undergoes recycling to the cell surface by binding to LRBA or is sorted to lysosome for degradation [81–84]. However, it is still unclear the mechanism of CTLA-4 autophagic degradation and the effect on cancer immunotherapy. In addition, autophagy induction increases antigens generation and T cell activation [20–23], while autophagy could degrade antigens in DCs or cancer cells leading to tumor immune escape (Fig. 5). However, autophagy deficiency or inhibition by using autophagy inhibitor chloroquine has no effect on T cell response in tumor-bearing mice [85]. Therefore, these controversial reports may be derived from different experimental context or models. For example, as an important regulator of autophagy, FIP200 inhibits AZI2/TBK1/IFN pro-inflammatory cytokine expressions, which could increase CD8^+^ T cell activity. However, in this process, FIP200 did not exhibit autophagy function [86]. Thus, it is necessary to determine the direct role of autophagy on cancer immune escape. Some autophagy-related genes could exhibit alternative function without autophagy induction. Moreover, autophagy could maintain the homeostasis of pro-inflammatory innate response [87], this study shows that selective autophagic degradation of TRIF by TAX1BP1 regulates the TRIF/TLR-induced robust pro-inflammatory immune response in macrophage. Some studies are in vitro analysis. Actually, in tumor microenvironment, autophagy regulates cancer immune escape will be more complicate. Therefore, autophagy maybe regulate the homeostasis of cancer immune response.

## Data Availability

Not applicable.

## Abbreviations

PD-L1: Programmed death ligand 1
PD-1: Programmed death 1
EMT: Epithelial-mesenchymal transition
GSK3β: Glycogen synthase kinase 3β
DHHC3: DHHC-type palmitoyltransferase
NSCLC: Non-small cell lung cancer
2-BP: 2-Bromopalmitate
APCs: Antigen-presenting cells
MHC: Major histocompatibility complex
M-MDSCs: Myeloid-derived suppressor cells
DCs: Dendritic cells
MVBs: Endosome-derived multivesicular bodies
MPR: Mannose-6-phosphate receptor
CTLA-4: Cytotoxic T-lymphocyte antige 4
LRBA: Lipopolysaccharide-responsive and beige-like anchor
